# Psychoneuroimmunomodulating effect of lymphocytes with ortho-fluoro-benzonal modulated activity in syngeneic long-term alcoholized recipients

**DOI:** 10.1192/j.eurpsy.2024.1420

**Published:** 2024-08-27

**Authors:** E. Markova, I. Savkin, E. Serenko, A. Smyk, M. Knyazheva

**Affiliations:** Neuroimmunology Lab, State Research Institute of Fundamental and Clinical Immunology, Novosibirsk, Russian Federation

## Abstract

**Introduction:**

Lymphocytes are dysfunctional during long-term ethanol consumption and may contribute the progression from healthy to problem drinking. GABAA receptors are molecular targets of ethanol on lymphocytes, potentiating the effects of alcohol.

**Objectives:**

We first demonstrated that original compound *ortho*-fluoro-benzonal, artificial GABA receptor ligand, has immunostimulating properties and is able to restored long-term alcoholized mice lymphocytes activity *in vitro* through GABAA receptors.  Based on the previous results we investigated effects of the *ex vivo ortho*-fluoro-benzonal modulated lymphocytes in recipients with experimental alcoholism.

**Methods:**

Male (CBAxC57Bl/6)F1 mice with 6-month 10% ethanol exposure were undergoing the transplantation of syngeneic long-term alcoholized mice lymphocytes, pretreated *in vitro* with *ortho*-fluoro-benzonal. Recipient’s ethanol consumption, parameters of the nervous and immune systems functional activities were estimated.

**Results:**

It was shown that lymphocytes modulated *ex vivo* with *ortho*-fluoro-benzonal after intravenous injection caused in syngeneic long-term alcoholized recipients ethanol consumption decrease and stimulation of behavioral activity in the “open field” test against the background of changes in the level of a number of cytokines in pathogenetically significant brain structures. Stimulation of humoral immune response, estimated by the relative number of antibody-forming spleen cells was also detected in recipients after lymphocytes transplantation. The injected immune cells were recorded in the parenchyma of the spleen and brain of recipients, which suggests, in particular, their direct influence on these functions.

**Conclusions:**

Results demonstrated that transplantation of *ortho*-fluoro-benzonal-modulated lymphocytes caused positive psychoneuroimmunomodulating effect in long-term alcoholized recipients, which makes it possible to consider adoptive immunotherapy as a promising method in the treatment of alcoholism.

**Disclosure of Interest:**

None Declared

